# Enhancement of capsular hypermucoviscosity in *Klebsiella pneumoniae* by *Acanthamoeba*

**DOI:** 10.1371/journal.pntd.0011541

**Published:** 2023-08-11

**Authors:** Jian-Ming Huang, Ko-Chiang Sung, Wei-Chen Lin, Hong-Yue Lai, Yu-Jen Wang

**Affiliations:** 1 School of Medicine, National Tsing Hua University, Hsinchu, Taiwan; 2 Institute of Molecular and Cellular Biology, National Tsing Hua University, Hsinchu, Taiwan; 3 Department of Clinical Laboratory, Chest Hospital, Ministry of Health and Welfare, Tainan, Taiwan; 4 Department of Microbiology and Immunology, College of Medicine, National Cheng Kung University, Tainan, Taiwan; 5 Department of Parasitology, College of Medicine, National Cheng Kung University, Tainan, Taiwan; 6 Department of Pharmacology, School of Medicine, China Medical University, Taichung, Taiwan; 7 Department of Parasitology, School of Medicine, China Medical University, Taichung, Taiwan; Chang Gung University, TAIWAN

## Abstract

**Background:**

*Acanthamoeba* and *Klebsiella pneumoniae* are both environmental commensals. Recently, clinical harm caused by hypermucoviscous *K*. *pneumoniae* has been observed. However, the interaction between these microbes and the origin of hypermucoviscous *K*. *pneumoniae* have not been reported

**Methodology/Principal findings:**

Here, we report that the bacterial capsule is enlarged when co-cultured with *Acanthamoeba* using India ink staining, and this effect depends on the number of parasites present. This interaction results in an enhancement of capsular polysaccharide production in the subsequent generations of *K*. *pneumoniae*, even without co-culturing with *Acanthamoeba*. The hypermucoviscosity of the capsule was examined using the sedimentation assay and string test. We also screened other *K*. *pneumoniae* serotypes, including K1, K2, K5, and K20, for interaction with *Acanthamoeba* using India ink staining, and found the same interaction effect

**Conclusions/Significance:**

These findings suggest that the interaction between *Acanthamoeba* and *K*. *pneumoniae* could lead to harmful consequences in public health and nosocomial disease control, particularly hypermucoviscous *K*. *pneumoniae* infections.

## Author summary

We investigated the interaction between the two environmental commensals *Acanthamoeba* and *Klebsiella pneumoniae*, and the origin of hypermucoviscosity in *K*. *pneumoniae*. Our study findings show that co-culturing *K*. *pneumoniae* with *Acanthamoeba* results in an enlarged bacterial capsule, leading to an increase in capsular polysaccharide production in the subsequent generations of *K*. *pneumoniae*. We screened several *K*. *pneumoniae* serotypes and observed the same effect. We demonstrated that the interaction between *Acanthamoeba* and *K*. *pneumoniae* could be a crucial factor in controlling hypermucoviscous *K*. *pneumoniae* infections.

## Introduction

*Klebsiella pneumoniae* is a gram-negative bacterium that mainly attacks hosts with a weakened immune system, causing opportunistic and multiple infections [1]. *K*. *pneumoniae* represents a high proportion of common pathogens in community pneumonia and nosocomial infection in Taiwan [2]. *K*. *pneumoniae* is an environmental commensal rather than only a clinical pathogen. The environmental habitats of *K*. *pneumoniae* include the human mucosal surfaces, soil, and surface water [3]. Recently, with the emergence of extremely aggressive *K*. *pneumoniae* strains, a new *K*. *pneumoniae* infection has been identified in Taiwan, which has been reported to have spread globally [4, 5]. These hypervirulent strains, which can occasionally cause pneumonia and lung abscesses, are the predominant isolates from patients with pyogenic liver abscesses. Based on the previous studies, these clinical stains of *K*. *pneumoniae* contain thickened capsule and increased mucoviscosity compared with those of classical strains (cKP) [6]. However, although many studies have investigated the capsular formation mechanism of hypermucoviscous *K*. *pneumoniae* (hvKP), the process of transformation of cKP into hvKP is not clear.

*Acanthamoeba*, a free-living amoeba (FLA), have been isolated from soil, water, and air, and frequently form organic biofilms [7]. They can growth private and public water systems, including those used by hospitals, homes, swimming pools, dental clinics, and cooling towers [8]. *Acanthamoeba* feed on bacteria, fungi, and other protists for nutrition; however, many microbes escape digestion by *Acanthamoeba* [9, 10]. In the microenvironment, *Acanthamoeba* secrete various proteins and metabolites, and in recent years, these extracellular substances have been found to have positive and negative effects on the pathogenicity of co-localizing microorganisms [11]. For instance, previous studies have revealed that certain secreted proteins, such as aminopeptidases and exosome-like vesicles, have been identified as virulence factors in pathogenic *Acanthamoeba* [11, 12]. These proteins, when released by *Acanthamoeba*, disrupt epithelial cells and induce apoptosis [13]. The extracellular proteins secreted by *Acanthamoeba* not only affect the amoeba itself but also influence the microbiota in the microenvironment [14, 15]. For instance of its location in the rhizosphere, which is crucial for plant-bacteria interactions and plant nutrient uptake [16].Amoeba treatment confirms the dominant role of bacterial grazers in shaping bacteria-plant interactions and promoting plant growth

Some microorganisms have evolved adaptive mechanisms that confer resistance against protists. These microorganisms have developed strategies to either avoid internalization by amoebae or to survive, multiply, and ultimately exit from free-living amoebae following engulfment. Previous studies have demonstrated that *Legionella pneumophila* possesses the ability to undergo intracellular multiplication within *Acanthamoeba* [17, 18]. By successfully evading or surviving within protists, these microorganisms demonstrate their capacity to adapt and thrive in the presence of amoebal predators. So far, there are only few studies that reported the extracellular effect of the interaction between *Acanthamoeba* and co-localizing bacteria on human health.

In this study, we examined the interaction between *Acanthamoeba* and *K*. *pneumoniae* in a co-cultured experiment, and India ink staining was used to analyze the phenotypic changes in *K*. *pneumoniae*. The size of capsule containing cell and the viscosity of capsular polysaccharide were determined. Moreover, four serotypes of *K*. *pneumoniae* isolates present in the environment were test to validated the effect of the interaction between parasites and bacteria. This research will help to underline the importance of microorganism’s interaction in the microenvironment and will give prominence to those related with public health and infection control.

## Materials and methods

### Ethics statement

The study protocol followed the Declaration of Helsinki criteria and was approved by the National Cheng Kung University Hospital’s Institutional Review Board in Tainan City, Taiwan. (Protocol code: B-ER-109-108; Date of approval: 8 June 2020). We have obtained formal written consent from the relatives of the donor who have agreed to participate in the research

### Culture of *Acanthamoeba* protozoa

The genotype T4 strain of *Acanthamoeba*, which was isolated from soil, was identified as *Acanthamoeba castellanii* strain Neff, ATCC-30010 and acquired from the American Type Culture Collection (ATCC, Manassas, VA, USA). ATCC-30010 was isolated from soil in Pacific Grove, California. *Acanthamoeba* cells were cultivated in protease peptone-yeast extract-glucose medium (20 g proteose peptone, 18 g glucose, 2 g Yeast extract, 1 g Sodium citrate dihydrate, 0.98 g MgSO4, 0.34 g KH2PO4, 0.188 g Na_2_HPO_4_ × 7H_2_O, 0.02 g Fe(NH4)2(SO4)2 × 6H2O in 1 liter ddH_2_O with pH 6.5) at 28°C in cell culture flasks, and they were washed and suspended in Page’s modified Neff’s amoeba saline (1.2 g NaCl, 0.04 g MgSO_4_-7H_2_O, 0.03 g CaCl2, 1.42 g Na_2_HPO_4_, 1.36 g KH_2_PO_4_ in 1 liter ddH_2_O) three times.

### Culture of Klebsiella pneumoniae

*K*. *pneumoniae* isolates were cultured in Luria-Bertani (LB) agar (solidified with 1.5% (w/v) agar) or LB broth. Clinical strains of *K*. *pneumoniae* were isolated from the corneal surface of hospitalized patients without any ocular disorder.

### Klebsiella pneumoniae stimulation by Acanthamoeba

To evaluate the interaction between *K*. *pneumoniae* and *Acanthamoeba*, we modified the direct contact method described in a previous study [19]. *K*. *pneumoniae* isolates were cultivated in LB broth at 37°C until they reached the exponential phase. The cultures were then harvested by centrifugation at 3000 rpm for 5 minutes and resuspended in 200 μL of phosphate-buffered saline (PBS) to obtain the desired experimental concentrations (OD_600_ = 0.2, 0.4, 0.8, 1.6), respectively. A calculated number of Acanthamoeba cells (4 × 10^4^, 5 × 10^4^, 8 × 10^4^, and 1.6 × 10^5^) were uniformly mixed with the bacterial cells in 200 μL of PBS. The mixture containing both bacterial and *Acanthamoeba* cells was incubated in a 24-well plate and placed in a thermostatic incubator for overnight incubation at 28°C.

### India ink staining

The application of India ink stain reveals the presence of capsules surrounding the organisms, which are visually identifiable as a halo [20]. Equal amounts of co-culture and India ink (Sigma-Aldrich, St. Louis, MO) were mixed on a glass slide and then covered with a coverslip. An OLYMPUS BX51 microscope (Olympus, Tokyo, Japan) was used at a magnification of 1000 ×.

### Size measurement of *Klebsiella pneumoniae* cells

After India ink staining, a total of 50 *K*. *pneumoniae* cells were screened under OLYMPUS BX51 microscope for measuring the diameter of dye-unpenetrated area. The GraphPad Prism 5.0 (La Jolla, CA, USA) was used to calculate and interpret statistical data using unpaired two-tailed Student’s *t*-test [21].

### Phenotype passage test

*K*. *pneumoniae* cells were co-cultured with *Acanthamoeba* for 24 h. The broth containing *Acanthamoeba* co-cultured *K*. *pneumoniae* was subcultured in LB broth using bacteriological loop and then passaged two times overnight at 37°C. The final passage bacterial colony was screened by India ink staining. Three independent experiments were performed, and images were captured.

### Sedimentation assay

Being hypermucoviscous, *K*. *pneumoniae* capsules do not sediment well after centrifugation. We have modified a sedimentation assay from the previous study [22]. Briefly, utilizing comparable bacterial culture quantities in 3 mL of LB broth, followed by a 10-min centrifugation at 3000 rpm. The optical density (OD) of the sedimentation assay’s supernatant was measured at a wavelength of 600 nm. Student’s *t*-test was used to determine statistical significance.

### Phenol-sulfuric acid assay

*K*. *pneumoniae* capsules containing uronic acid were subjected to the phenol-sulfuric acid assay using previously described techniques [23, 24]. Extracts from similar amounts of overnight bacterial cultures were reconstituted in 0.1 mL of water, and mixed with 1.2 mL of 12.5 mM tetraborate in concentrated H_2_SO_4_. The liquid was vigorously vortexed before being allowed to boil for 5 min. After cooling, 20 mL of 0.15% 3-hydroxydiphenol (Sigma-Aldrich, St. Louis, MO) was added, and absorbance at 520 nm was measured. Student’s *t*-test was used to determine statistical significance.

### String test

*K*. *pneumoniae* isolates co-cultured with or without *Acanthamoeba* were assessed for the hypermucoviscous phenotype using the string test. On blood agar plates, each isolate was grown overnight at 37°C before expansion of bacterial colonies using an inoculation loop. A positive test result is determined when bacterial colonies stretched on an agar plate with a bacteriology inoculation loop form a viscous string measuring more than 5 mm in length as previous described [25].

### Identification of *Klebsiella pneumoniae* serotype

The serotyping was performed on the *wzc* sequencing according to a previous study [26]. Briefly, after *K*. *pneumoniae* DNA extraction, two primer pairs were used to amplify the consensus sequence, including primer pair 1: KP-*wza*-CF1 and KP-*wzc*-CR1, and primer pair 2: KP-*wza*-CF2 and KP-*wzc*-CR2. The cycling program consisted of 96°C for 3 min, followed by 30 temperature cycles at 96°C for 30 s, 46°C for 15 s, and 72°C for 3 min. After DNA amplification, the products with the predicted size of 2.7 kb (primer pair 1) and 3.1 kb (primer pair 1) were obtained, and nBLAST tool and National Center for Biotechnology Information (NCBI) database were used.

### Isolation of environmental *Klebsiella pneumoniae*

Samples of water were gathered from canals and rivers located within a 20-kilometer radius of Taichung City. Each sample was filtered through a 0.45 mm-diameter filter (Sartorius, Göttingen, Germany). The membranes were placed on Simmons citrate agar with 1% (w/v) inositol and incubated for 48 h at 37°C. The recovery of *Klebsiella* is not inhibited but is very selective in this medium [27]. Isolation of potential *Klebsiella* colonies was followed by identification using the biochemical tests, including fermentation of melezitose and L-sorbose, gas production from lactose at 44.5°C, growth at 10°C, pectate degradation, and utilization of m-hydroxybenzoate and hydroxy-L-proline.

## Results

**Fig 1:**
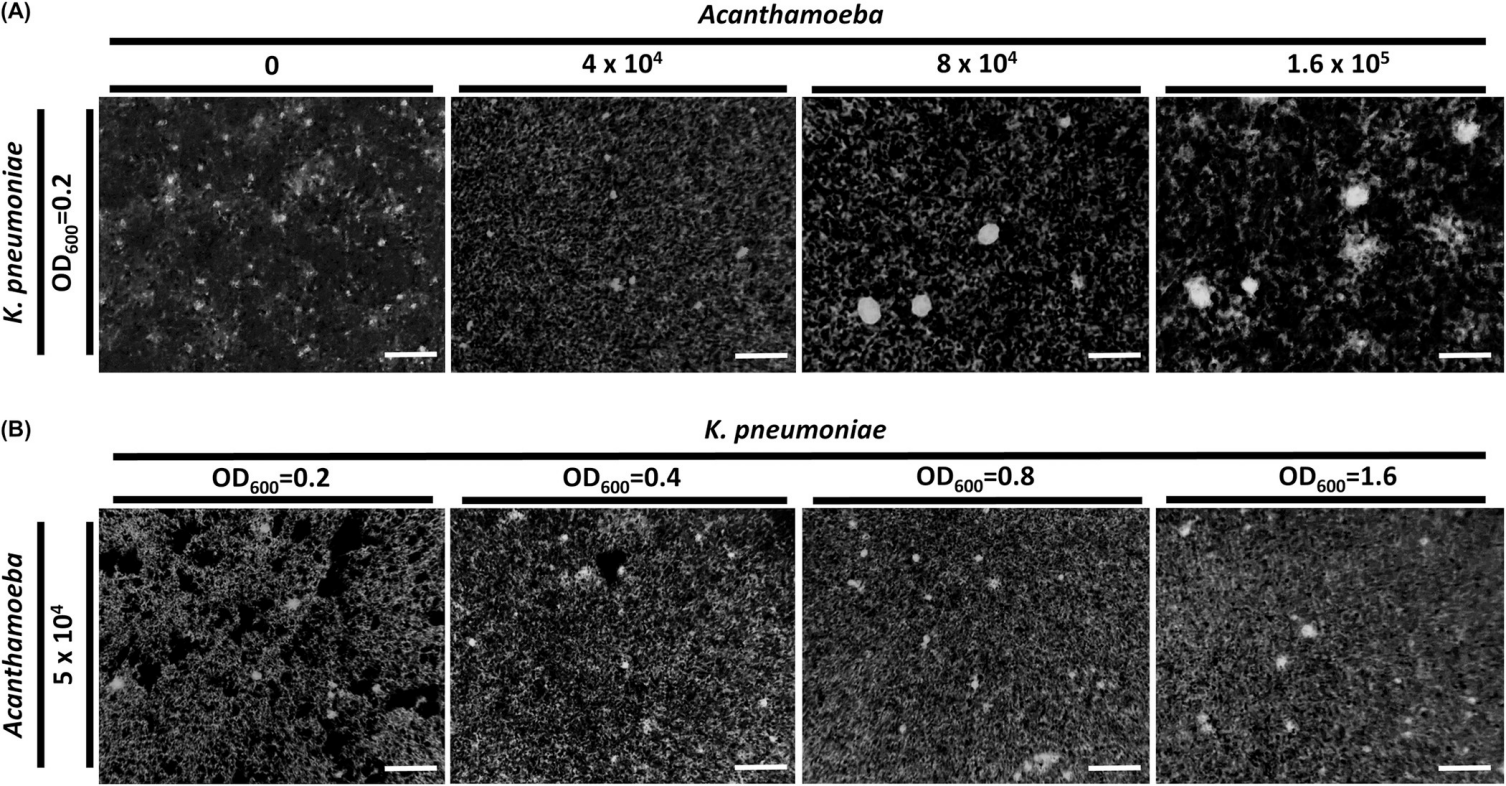
India ink staining of *K*. *pneumoniae* cells from overnight co-culturing at serial density. **(A).** *Acanthamoeba* cells at a density of 4 × 10^4^, 8 × 10^4^, and 1.6 × 10^5^ were overnight co-cultured with *K*. *pneumoniae* adjusted at OD_600_ value of 0.2. **(B).** *K*. *pneumoniae* cells at OD_600_ of 0.2, 0.4, 0.8, and 1.6 were overnight co-cultured with *Acanthamoeba* cells at a density of 5 × 10^4^ for India ink staining. Scale bar = 10 μm.

To investigate the impact of the interaction between *Acanthamoeba* and *Klebsiella pneumoniae*, *Acanthamoeba* cells at different calculated numbers were co-cultured with clinical *K*. *pneumoniae* isolates adjusted to a consistent optical density. Simultaneously, *K*. *pneumoniae* cells, adjusted to various experimental densities, were co-cultured with *Acanthamoeba* cells at a fixed count. After overnight co-culturing, *K*. *pneumoniae* cells were harvested using India ink staining. *K*. *pneumoniae* cells containing capsule prevent large particles of dye to penetrate the cell and thus provide a negative background for analysis. Interestingly, *K*. *pneumoniae* capsules were visually enlarged after co-culturing with *Acanthamoeba* cells at a density of 8 × 10^4^ and 1.6 × 10^5^ but showed similar size with the cells at 4 × 10^4^ compared with *K*. *pneumoniae* without *Acanthamoeba* as negative control (Fig 1A). However, *Acanthamoeba* cells at a density of 5 × 10^4^ could not stimulate *K*. *pneumoniae* capsule to be enlarged at either of the OD_600_ value of 0.2, 0.4, 0.8, or 1.6 (Fig 1B). India ink staining data showed that *Acanthamoeba* could affect *K*. *pneumoniae* in the microenvironment, resulting in enlargement of *K*. *pneumoniae* cells containing capsule depending on the numbers of *Acanthamoeba* cells but not on the density of *K*. *pneumoniae* cells.

**Fig 2:**
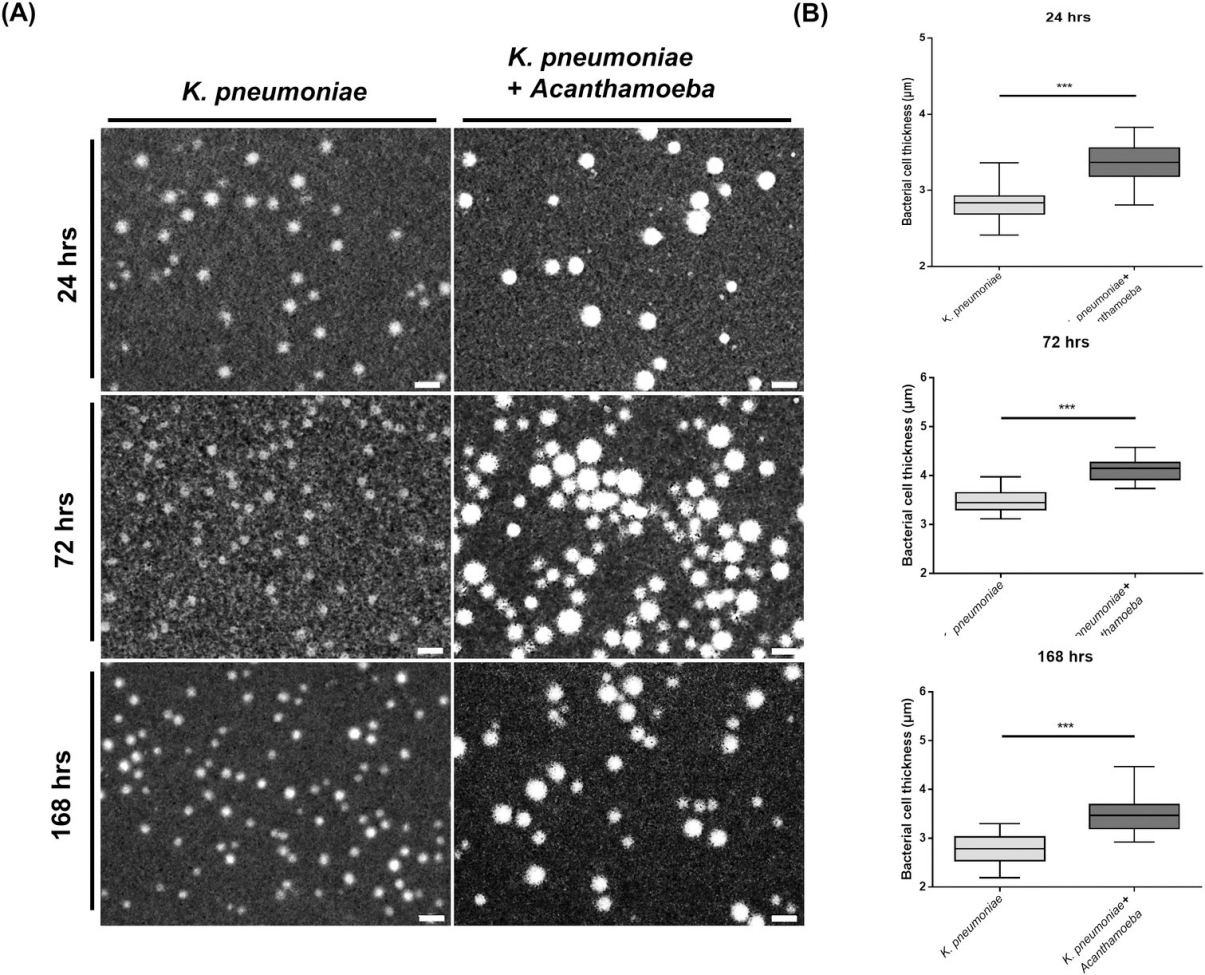
Measurement of *K*. *pneumoniae* cell size using India ink staining after co-culturing with *Acanthamoeba*. **(A).** India ink staining images of *K*. *pneumoniae* at a magnification of 1000× after co-culturing with *Acanthamoeba*. Bacterial cells were screened after co-culturing with *Acanthamoeba* at 24, 72, and 168 h. Scale bar = 10 μm. **(B).** Comparison of average cell size between co-cultured and cultured alone *K*. *pneumoniae* at 24, 72, and 168 h. The average cell size was calculated for 50 bacterial cells observed under a magnification of 1000×. Statistical significance was determined by Student’s *t*-test. ***P < 0.001.

Furthermore, the size of enlarged *K*. *pneumoniae* capsule was analyzed. *Acanthamoeba* cells at a density of 1.0 × 10^5^ were added into the microenvironment containing *K*. *pneumoniae* cells adjusted to an OD_600_ of 0.2 for 24, 72, and 168 h. After the end of experiment, *K*. *pneumoniae* cells incubated alone or co-cultured with *Acanthamoeba* were harvested for cell size measurement. The capsules of *K*. *pneumoniae* cells were then stained with India ink, and microscopic images of 50 individual cells were captured for size measurement. The size was measured as the diameter of dye-unpenetrated area of the cell containing the capsule surrounding the bacterial cell. *K*. *pneumoniae* cells cultured alone had an average capsular size of 2.827, 3.511, and 2.798 μm after 24, 72, and 168 h, respectively. By contrast, *K*. *pneumoniae* cells co-cultured with *Acanthamoeba* had an average capsular size of 3.346, 4.086, and 3.454 μm after 24, 72, and 168 h, respectively, indicating significant increase at all time intervals (Fig 2). The data suggested that *Acanthamoeba* could continually stimulate enlargement of the capsule size around *K*. *pneumoniae* cells.

**Fig 3:**
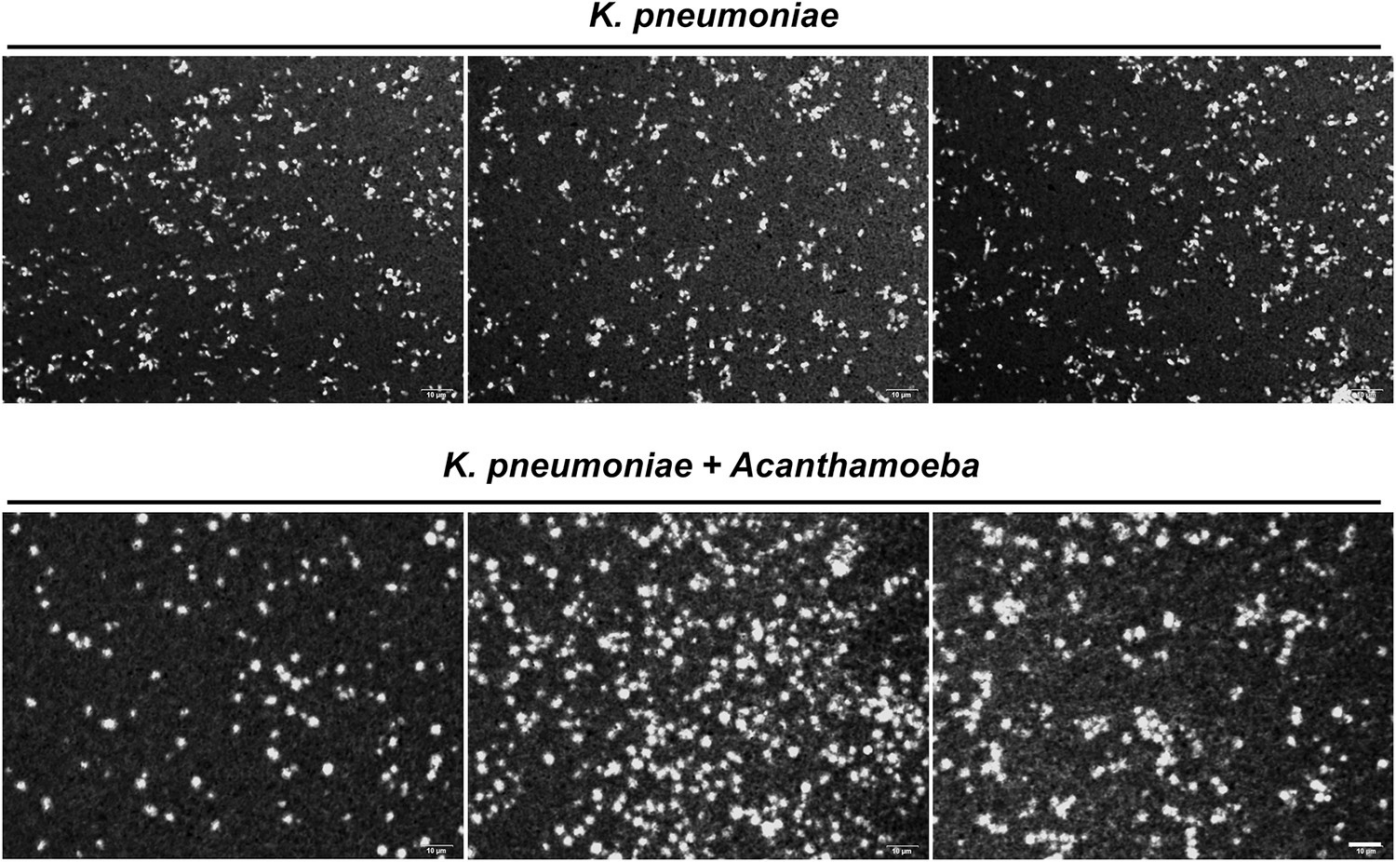
Images of *K*. *pneumoniae* cells passaged two times after culturing alone or co-culturing with *Acanthamoeba*. *Acanthamoeba* co-cultured *K*. *pneumoniae* or *K*. *pneumoniae* cultured alone was passaged two times in Luria-Bertani broth. The third generation of *K*. *pneumoniae* cells were harvested using India ink staining. The images were captured from three independent experiments.

However, whether the impact of *Acanthamoeba* presence on the capsule size of *K*. *pneumoniae* cells was brief or persistent would have importance in infection control. Hence, *Acanthamoeba* co-cultured *K*. *pneumoniae* cells were harvested and then passaged for two times without *Acanthamoeba*. The passaged *K*. *pneumoniae* cells were compared for the capsule size using India ink staining. Surprisingly, *Acanthamoeba* co-cultured *K*. *pneumoniae* cells passaged two times showed enlarged capsule compared with *K*. *pneumoniae* cultured alone (Fig 3). The presence of enlarged capsule of *K*. *pneumoniae* in cells passaged two times without *Acanthamoeba* indicated the outcome of microorganism’s interaction was critical for the follow up problem in public health.

**Fig 4:**
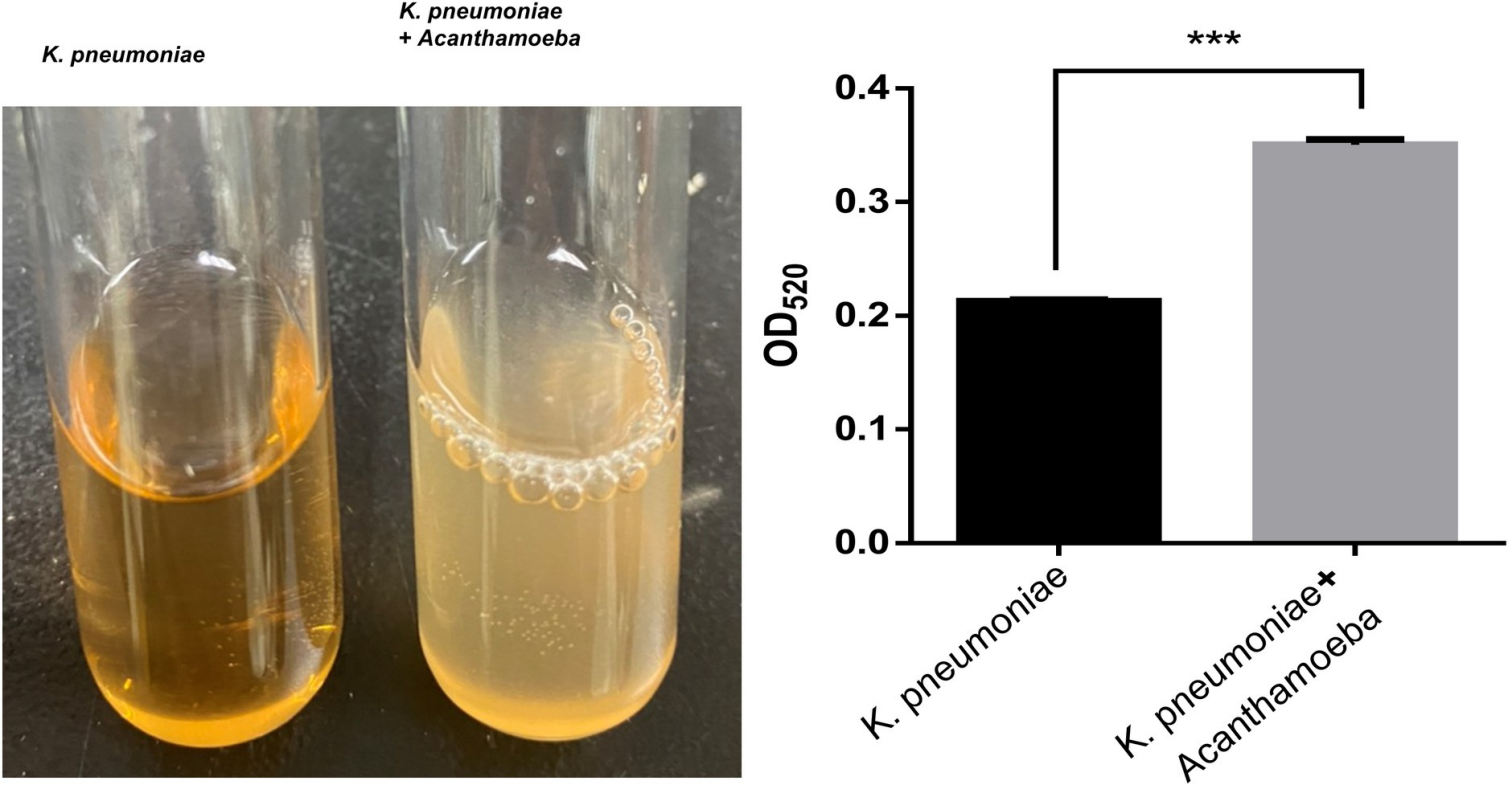
Phenol-sulfuric acid assay for capsular polysaccharide of *K*. *pneumoniae*. The cells from *K*. *pneumoniae* cultured alone or *Acanthamoeba* co-cultured *K*. *pneumoniae* were treated with sulfuric acid. Uronic acid from *K*. *pneumoniae* capsular polysaccharide was identified as an orange complex at a wavelength of 520 nm after phenol treatment. Statistical significance was determined by Student’s *t*-test. ***P < 0.001.

Based on significant enlargement in capsule size of *K*. *pneumoniae* after stimulating by *Acanthamoeba*, we further validated the capsule containing *K*. *pneumoniae* cell using phenol-sulfuric acid assay to quantify the capsular polysaccharide. The polysaccharide layer of *K*. *pneumoniae* capsule extends outside the cell envelope, and uronic acid-based acid complex carbohydrates make up a sizable component of the polysaccharide conjugates. Uronic acid was detectable as an orange compound at a wavelength of 520 nm following phenol treatment, which followed sulfuric acid’s conversion of the polysaccharide to monosaccharide. As a result, *Acanthamoeba* co-cultured *K*. *pneumoniae* had a more complex chemical makeup and appeared cloudy during cell diameter measurement. The abundance of the capsular polysaccharide in *Acanthamoeba* co-cultured *K*. *pneumoniae* was shown by considerably higher absorption (OD_520_ = 0.351) of the strain than that of *K*. *pneumoniae* cultured alone (OD_520_ = 0.213) (Fig 4). The data from phenol-sulfuric acid assay confirmed that *Acanthamoeba* enhanced the capsular polysaccharide secretion in *K*. *pneumoniae*.

**Fig 5:**
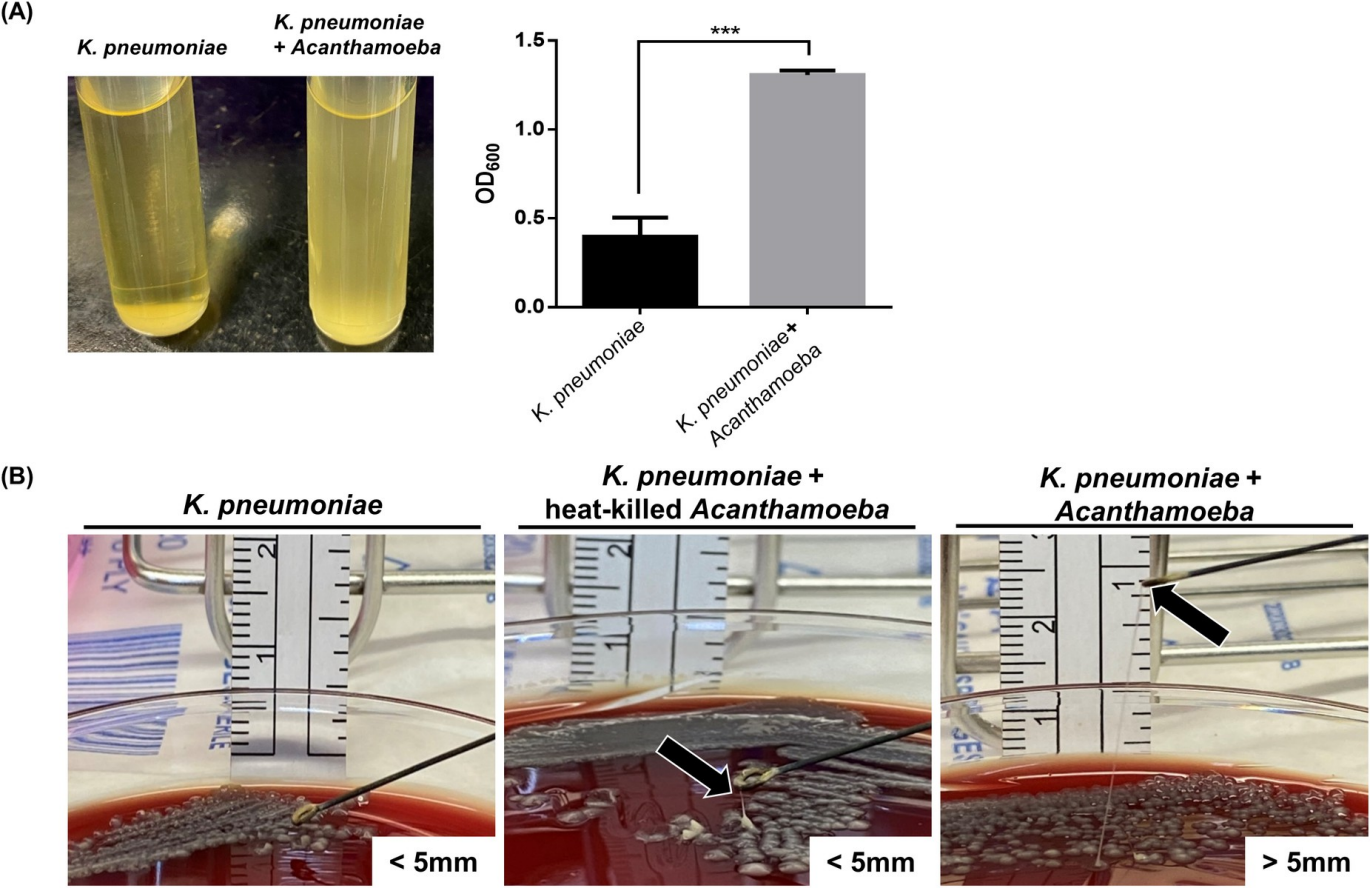
*K*. *pneumoniae* capsular viscosity examination by sedimentation assay and string test. **(A).** The cells from *K*. *pneumoniae* cultured alone or *Acanthamoeba* co-cultured *K*. *pneumoniae* were incubated overnight in Luria-Bertani broth. The broth was centrifuged at 3000 rpm for 10 min. The absorbance of the supernatants was measured at 600 nm. Statistical significance was determined by Student’s *t*-test. ***P < 0.001. **(B).** Comparison of stretched colonies between *K*. *pneumoniae* cultured alone and *Acanthamoeba* co-cultured *K*. *pneumoniae*. A positive string and hypermucoviscous phenotype were defined by strings 5 mm in length or longer. The black arrows indicate the top of the string; however, no stretched string was observed for *K*. *pneumoniae* cultured alone.

Furthermore, the enlarged capsule of *K*. *pneumoniae* cell containing more polysaccharide would be important for clinical treatment of *K*. *pneumoniae* infection. Also, bacterial viscosity of *K*. *pneumoniae* is often associated with pathogenicity. Therefore, sedimentation assay and string test were performed to quantify the capsule viscosity. Sedimentation assay revealed that the supernatant of *Acanthamoeba* co-cultured *K*. *pneumoniae* was viscous, and that the precipitation of bacterial cells was difficult. The supernatant of *Acanthamoeba* co-cultured *K*. *pneumoniae* (OD_600_ = 1.307) had higher viscosity than that of *K*. *pneumoniae* cultured alone (OD_600_ = 0.397) (Fig 5A). The string test was used to identify pathogenic characteristic of hypermucoviscous phenotype of *K*. *pneumoniae*, and it is a standard method to test the hypervirulent *K*. *pneumoniae* in clinical laboratory. When a loop is used to stretch the colony on an agar plate, a positive string test is one that shows the development of viscous strings >5 mm in length. Therefore, we used the string test to examine the bacterial colonies of *K*. *pneumoniae* cultured alone, live *Acanthamoeba* co-cultured *K*. *pneumoniae*, and heat-killed *Acanthamoeba* co-cultured *K*. *pneumoniae*. Live *Acanthamoeba* co-cultured *K*. *pneumoniae* exhibited a bacterial strings > 5 mm in length. However, bacterial string from the colony of either heat-killed *Acanthamoeba* co-cultured *K*. *pneumoniae* or *K*. *pneumoniae* cultured alone presented a length of <5 mm (Fig 5B). The viscosity data of *K*. *pneumoniae* from sedimentation assay and string test showed that *Acanthamoeba* induced hypermucoviscosity in *K*. *pneumoniae* cells, and clinical treatment should be developed based on these findings.

**Fig 6:**
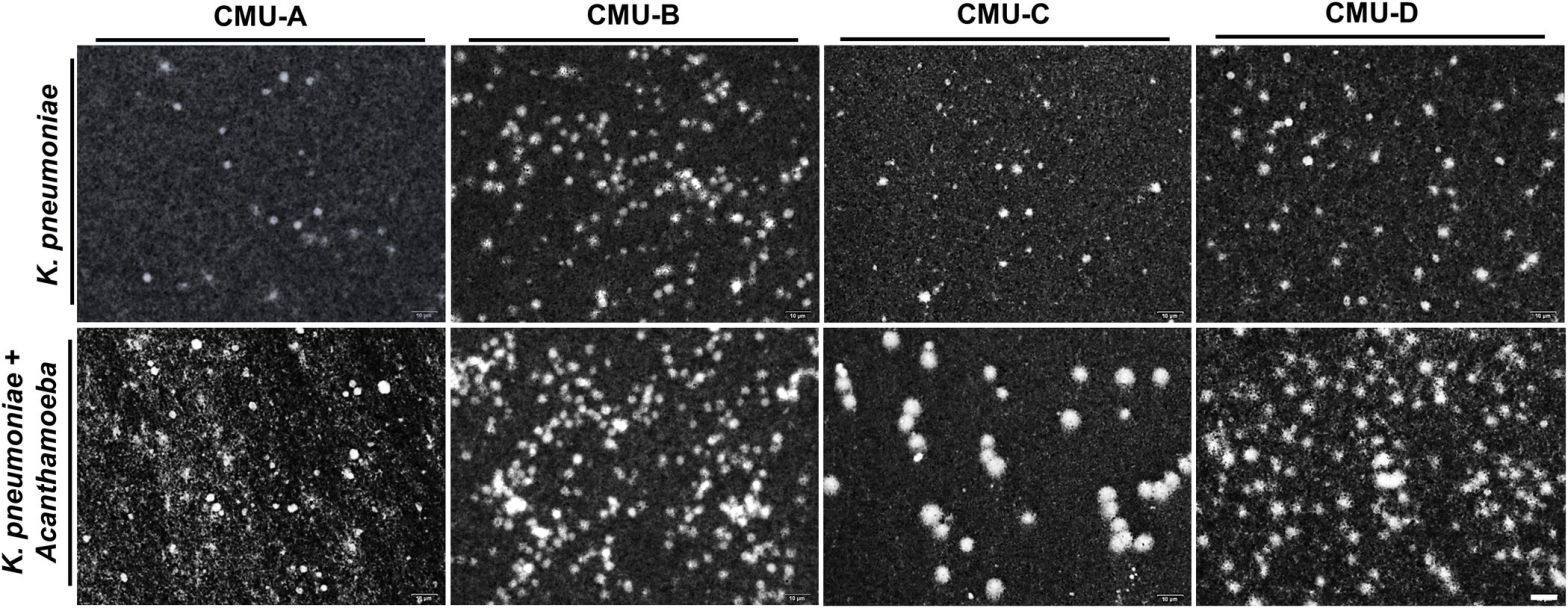
India ink staining of various serotypes *K*. *pneumoniae* environmental isolates treated with *Acanthamoeba*. The images of *Acanthamoeba* co-cultured *K*. *pneumoniae* environmental isolates or isolates cultured alone after staining with India ink at a magnification of 1000×. The serotypes of the environmental isolates are as follows: CMU-A belongs to serotype K1, CMU-B belongs to serotype K2, CMU-C belongs to serotype K5, and CMU-D belongs to serotype K20. Scale bar = 10 μm.

Finally, we used the *wzc* sequencing to identify the serotype of *K*. *pneumoniae* and collect several different serotypes of *K*. *pneumoniae* to validate the stimulation of capsule secretion by *Acanthamoeba*. According to the *wzc* sequencing, the original tested *K*. *pneumoniae* belong to the K81. Simultaneously, the serotypes K1, K2, K5, and K20 were also collected from the environment to test the microorganism’s interaction with *Acanthamoeba*. Obviously, the effect of *Acanthamoeba* on *K*. *pneumoniae* capsule secretion was observed after treatment with *Acanthamoeba* for 24 h using India ink staining (Fig 6). India ink staining data of *Acanthamoeba* treatment of diverse serotypes of *K*. *pneumoniae* indicated that enhancement of *K*. *pneumoniae* capsule secretion by the interaction with *Acanthamoeba* in the microenvironment was extensive, and that would be crucial for public health and infection control.

## Discussion

The existence of diverse endosymbiotic bacteria within *Acanthamoeba* hosts has been well-established for a significant duration [28]. Previous study showed that clinical isolates of *Acanthamoeba* harbored several medically important bacteria including *Legionella*, *Mycobacterium*, *Pseudomonas*, *Malassezia* and *Chlamydia* [29, 30]. It is noteworthy that these bacteria enable them to utilize *Acanthamoeba* as a training ground to further evade the challenges of the microenvironment, which in turn impacts human infections. For instance, *L*. *pneumophila* cells cultivated in amoebae demonstrated a minimum of 100-fold higher invasiveness towards epithelial cells and a 10-fold higher invasiveness towards macrophages and *Acanthamoeba* [31]. Furthermore, it has recently been discovered that *Campylobacter jejuni*, which is sensitive to environmental stresses, has the ability to invade amoebae, and the invaded cells still undergo encystation [32]. These studies collectively indicate that the interaction between *Acanthamoeba* and bacteria is an important and significant research topic concerning human health hazards.

In this study, we revealed that *Acanthamoeba* could influence *K*. *pneumoniae* capsular polysaccharide secretion in the microenvironment. While the quantity of *Acanthamoeba* tested in this study may not necessarily be highly prevalent in aquatic environments, the proportional calculation based on the amount of co-cultured *K*. *pneumoniae* suggests that a significant proportion of *Acanthamoeba* populations is still likely to occur in various water bodies, such as rivers, farmlands water, and water towers [33]. If we neglect its presence, it could potentially lead to an increase in its population. It is important to note that population densities of *Acanthamoeba* can vary greatly across different habitats, including freshwater bodies, soil, and man-made environments. Factors such as nutrient availability, temperature, and the presence of suitable hosts can influence the abundance of *Acanthamoeba* in the wild [34]. Therefore, the range mentioned in our study serves as a representative estimate rather than an absolute value applicable to all natural settings.

The enhancement of *K*. *pneumoniae* capsular polysaccharide production was dependent on the number of *Acanthamoeba* cells, suggesting that the effect was as a result of extracellular stimulation by *Acanthamoeba*. Previous studies have reported that several enzymes or metal ions are released from *Acanthamoeba* to the microenvironment. The function of potassium ion transporters in the sensory perception of the *Acanthamoeba* T4 genotype was discovered by Siddiqui et al [35]. Also, proton pump, sodium and calcium transporters, and others have all been shown to have a role in the amoeba’s differentiation [36]. Besides, to survive in the environment and infect the host, *Acanthamoeba* releases a number of metal-containing enzymes. Iron superoxide dismutase (Fe-SOD) released by *Acanthamoeba* assist amoeba cells in preventing the environmental oxygen stress. Moreover, a 38-kDa copper-zinc SOD (Cu-Zn-SOD) was isolated from *Acanthamoeba* to demonstrate its antioxidant and anti-inflammatory properties. Hence, the concentration of microenvironmental metal ions change based on *Acanthamoeba*’s survival, which might accidentally interfere with the phenotype of surrounding microorganisms. Recently, a hypermucoviscous strain of *K*. *pneumoniae* (hvKP) has spread across the world. The community can contract illnesses from hvKP as it is more virulent than that of cKP and frequently affects healthy individuals.

Owing to one of the most frequent causes of hospital acquired infections (HAI) and rise in antibiotic-resistant strains, *K*. *pneumoniae* has become a significant public health concern. This opportunistic pathogen typically colonizes the gut, throat, and nasal passages without spreading disease, but it can sometimes spread infections to the lungs, skin, urinary system, and bloodstream [37]. The hvKP is now recognized for its propensity to cause a number of illnesses. It was first shown to be a cause of pyogenic liver abscesses in Asia [38]. However, uncertainty regarding the origin of hvKP is a major problem in investigations. Coincidentally, the opportunistic pathogen in HAI was also thought to be free-living *Acanthamoeba*. They have been identified in approximately 10.5% of amoeba-associated nosocomial illnesses and have been detected in water samples from 68.9% of hospital faucets [39, 40]. Moreover, *Acanthamoeba* was present in 26% of catheter urine samples from a critical care unit in a Brazilian hospital [41]. Therefore, the interaction between *K*. *pneumoniae* and *Acanthamoeba* can occur not only in the community but also in the nosocomial environment.

According to the data in this study, the polysaccharide level in *K*. *pneumoniae* capsule was elevated, resulting in hypermucoviscous *K*. *pneumoniae* with four distinct types of virulence factors, all of which have been extensively studied. Notably, hypervirulent bacteria are particularly hyperactive in terms of capsule production, siderophores, lipopolysaccharide (LPS), and fimbriae/pili. The structure known as the capsule encloses the bacteria and conforms to the polysaccharide to increase pathogenicity of *K*. *pneumoniae* [42]. The hypermucoviscous capsule had greater immunological resistance than that of the conventional capsule because of the presence of exopolysaccharides with high mucoviscosity. Several important genes for capsule formation are present in the operon *cps* on the bacterial chromosome of both conventional and high pathogenic *K*. *pneumoniae* strains [43]. The important capsule biosynthesis genes *wzi*, *wza*, *wzb*, *wzc*, *gnd*, *wca*, *cpsB*, *cpsG*, and *galF* are located at the *cps* gene cluster [26]. Furthermore, multiple studies have demonstrated that upregulating the expression of two plasmid-encoded transcriptional regulators, namely *rmpA* and *rmpA2*, or manipulating the RCS two-component system genes, *rcsA* and *rcsB*, can enhance the biosynthesis of the hypermucoviscous *K*. *pneumoniae* capsule [44, 45]. Hence, whether the extracellular stress or secreted substances from *Acanthamoeba* directly or indirectly stimulates the capsule biosynthesis genes of *K*. *pneumoniae* needs further investigation.

In conclusion, cKP was transformed into hvKP by interacting with *Acanthamoeba* in the microenvironment. The phenotypic changes in *K*. *pneumoniae* was not dependent on the number of bacterial cells but that of the parasite. Also, the bacterial capsule was enlarged with the time of co-culturing with *Acanthamoeba*. *Acanthamoeba*-stimulated *K*. *pneumoniae* could continually present hypermucoviscous capsule containing abundant polysaccharides. The parasite could interfere with the *K*. *pneumoniae* serotypes K1, K2, K5, K20, and K81, indicating an extensive microorganism’s interaction in the community or nosocomial environment. According to a previous study [46], we can utilize in silico molecular modeling investigations, molecular docking, molecular dynamics simulations, and computational toxicity assessments to understand the molecular mechanism underlying the interaction between the parasite and bacteria, taking advantage of advancements in computational approaches. In the future studies, we will constantly investigate the mechanisms underlying the interaction between *K*. *pneumoniae* and *Acanthamoeba*, and its impact on the public health and infection control.
